# Genetic variation of biomass recalcitrance in a natural *Salix viminalis* (L.) population

**DOI:** 10.1186/s13068-019-1479-7

**Published:** 2019-06-03

**Authors:** Jonas A. Ohlsson, Henrik R. Hallingbäck, Mohamed Jebrane, Anne E. Harman-Ware, Todd Shollenberger, Stephen R. Decker, Mats Sandgren, Ann-Christin Rönnberg-Wästljung

**Affiliations:** 1 Department of Molecular Sciences, Swedish University of Agricultural Sciences, Uppsala, Sweden; 2 Department of Plant Biology, Uppsala BioCenter, Linnean Centre for Plant Biology, Swedish University of Agricultural Sciences, P.O. Box 7080, 750 07 Uppsala, Sweden; 3 Department of Plant Physiology and Forest Genetics, Swedish University of Agricultural Sciences, Umeå, Sweden; 4 Department of Forest Biomaterials and Technology/Wood Science, Swedish University of Agricultural Sciences, Box 7008, 750 07 Uppsala, Sweden; 5 Biosciences Center, National Renewable Energy Laboratory, Golden, CO USA; 6 Center for Bioenergy Innovation, National Renewable Energy Laboratory, Golden, CO USA

**Keywords:** *Salix viminalis*, Bioenergy crops, Lignocellulosic biofuels, Genetic parameters, Genomewide association study, Enzymatic saccharification, Biomass recalcitrance, Plant breeding

## Abstract

**Background:**

*Salix* spp. are high-productivity crops potentially used for lignocellulosic biofuels such as bioethanol. In general, pretreatment is needed to facilitate the enzymatic depolymerization process. Biomass resistance to degradation, i.e., *biomass recalcitrance*, is a trait which can be assessed by measuring the sugar released after combined pretreatment and enzymatic hydrolysis. We have examined genetic parameters of enzymatic sugar release and other traits related to biorefinery use in a population of 286 natural *Salix viminalis* clones. Furthermore, we have evaluated phenotypic and genetic correlations between these traits and performed a genomewide association mapping analysis using a set of 19,411 markers.

**Results:**

Sugar release (glucose and xylose) after pretreatment and enzymatic saccharification proved highly variable with large genetic and phenotypic variations, and chip heritability estimates (*h*^2^) of 0.23–0.29. Lignin syringyl/guaiacyl (S/G) ratio and wood density were the most heritable traits (*h*^2^ = 0.42 and 0.59, respectively). Sugar release traits were positively correlated, phenotypically and genetically, with biomass yield and lignin S/G ratio. Association mapping revealed seven marker–trait associations below a suggestive significance threshold, including one marker associated with glucose release.

**Conclusions:**

We identified lignin S/G ratio and shoot diameter as heritable traits that could be relatively easily evaluated by breeders, making them suitable proxy traits for developing low-recalcitrance varieties. One marker below the suggestive threshold for marker associations was identified for sugar release, meriting further investigation while also highlighting the difficulties in employing genomewide association mapping for complex traits.

**Electronic supplementary material:**

The online version of this article (10.1186/s13068-019-1479-7) contains supplementary material, which is available to authorized users.

## Background

Transportation fuels produced from woody energy crops or other lignocellulosic biomass represent an important opportunity for increasing the renewable fraction of the energy supply without competing for resources otherwise used for food and feed purposes [1]. The bulk of energy in such feedstocks is contained within the cellular matrix of the secondary cell walls, which can be divided into three components: cellulose, a polymer of β-1,4 linked glucose residues; hemicelluloses, noncellulosic polysaccharides which in hardwoods are predominantly xylans; and lignin, a heterogeneous and highly aromatic polymer which envelops and bonds with the polysaccharides. Lignocellulosic biomass feedstocks are, however, more resistant to degradation than their food-grade counterparts. Microbial conversion of lignocellulosic biomass typically requires pretreatment and enzymatic hydrolysis steps in order to release the sugars, most commonly glucose but for some systems also xylose. Resistance to these processes is commonly referred to as *biomass recalcitrance* [2].

Biomass recalcitrance is a key barrier to the financial feasibility of lignocellulosic ethanol [2]. Recalcitrant feedstocks yield fewer fermentable carbohydrates per unit of energy used for pretreatment, may require higher doses of enzymes, and typically form more fermentation inhibitors due to the harsher pretreatment required. The recalcitrance of a feedstock is influenced by a multitude of factors including the composition and structure of the secondary cell wall polymers, and physical features affecting cellulose accessibility [3]. Recent improvements in high-throughput screening of recalcitrance have enabled the survey of large plant populations in the pursuit of low-recalcitrance varieties [4].

Although biomass recalcitrance is known to be highly multifactorial, lignin composition is believed to play an important role. Hardwood lignin, while highly heterogeneous in structure, is composed mainly of sinapyl alcohol (S) and coniferyl alcohol (G) monolignols. The molar ratio of these subunits within the lignin polymer, commonly referred to as the S/G ratio, influences several properties of the lignin polymer including the content of labile ether bonds, molecular weight, and linearity, but the correlation between S/G ratio and recalcitrance is not consistently and unidirectionally reported in the literature [5]. While direct screening of recalcitrance via pretreatment and saccharification assays can be regarded as the standard for recalcitrance on the basis of fermentable carbohydrate yield, such assays are labor-intensive even in high-throughput incarnations and requires large investments in equipment and infrastructure. In contrast, biomass composition can be evaluated inexpensively using minimal manpower and relatively available pyrolysis-mass spectrometry (py-MBMS) equipment and at the very least provides an indication of lignin composition but with further studies may also elucidate relationships with other biomass composition and properties [4, 6, 7].

Producing plant varieties better suited for biorefining can be accomplished through different breeding methods and strategies, such as genetic modification (GM) or by recurrent selection for objective biorefining traits or for other traits genetically correlated to such traits. Due to reasons of legislation and public perception, the recurrent selection strategy may be desirable in many cases. To speed up the selection process, marker-assisted selection (MAS) can be used in the early stages of a breeding program. Increasingly, markers are generated using genomewide association studies (GWAS), where large numbers of molecular markers such as single-nucleotide polymorphisms (SNPs) and insertions or deletions (indels) are evaluated together with the traits of interest. GWAS methodology together with improvements in genetic sequencing outputs and costs have enabled genetic surveys of marker–trait associations in large populations of unrelated individuals, enabling identification of interesting alleles in natural populations, which harbor considerable genetic variation [8].

Willows (*Salix* spp.), commonly grown in short-rotation coppice (SRC) systems as dedicated energy crops, represent a promising biorefinery feedstock. In an SRC system, the shoots are harvested after just a few years of growth, leaving the stools in the ground. In the following spring, new shoots emerge and the SRC cycle starts over. As willows are outcrossing, easily hybridized, readily clonally propagated through cuttings, and harbor considerable inter- and intraspecies genetic variabilities, they lend themselves well to breeding [9–11]. Furthermore, the short generation time (often flowering already in the first year) enables relatively rapid breeding cycles. In Europe, *S. viminalis* is among the most common *Salix* species used for breeding, owing to its high biomass yield potential [9, 12].

In *Salix*, the effect of genotype on enzymatic hydrolysis yields has been documented [13–15]. Brereton et al. [13] evaluated enzyme-derived glucose in a quantitative trait locus (QTL) mapping population, although the use of nonpretreated biomass may mean that its results are not applicable to pretreated biomass. Ray, Brereton, and coworkers [14] quantified sugar release after dilute acid pretreatment in 35 commercial *Salix* clones, finding considerable differences in recalcitrance among the included clones. By estimating potential ethanol yield on a liter per hectare basis, the authors found an approximately threefold difference between the best and worst performers, highlighting the importance of clone development for biorefining. Similar results were reported by Serapiglia et al. [15], in a study investigating sugar yields from ten different cultivars using hot-water pretreatment at two different severities. In the latter study, near-perfect ethanol yields were recorded for several clones at the higher severity, while there was considerable variation between clones at the milder pretreatment condition.

Taking the above findings into consideration, it is expected that there is a genetic basis of biomass recalcitrance in *Salix*, suggesting that surveying natural populations may be a viable strategy for identifying genes and genotypes contributing to reduced recalcitrance. Therefore, the current study was performed with the aim of assessing the variation in a number of biomass and wood traits, primarily related to enzymatic sugar release, in a population of *S. viminalis* genotypes collected throughout Europe and Russia. Furthermore, the genetic background of these phenotypic traits was analyzed using a large set of genomewide markers.

## Results

From each plant of the 291 vegetatively replicated accessions (i.e., clones) from four blocks, the main shoot was harvested. Excluding dead and damaged plants, 1132 individual shoots remained for subsequent analyses, amounting to an average of 3.57 biological replicates per clone, with 286 clones remaining. Shoots that were too small to be chipped were further excluded from the sugar release assay (*n* = 1097 for the sugar release assay), and shoots that were deemed too small to accurately measure the densities of were excluded from such measurements (*n* = 1109 for density measurements).

### Phenotypic and genetic variation

**Fig. 1:**
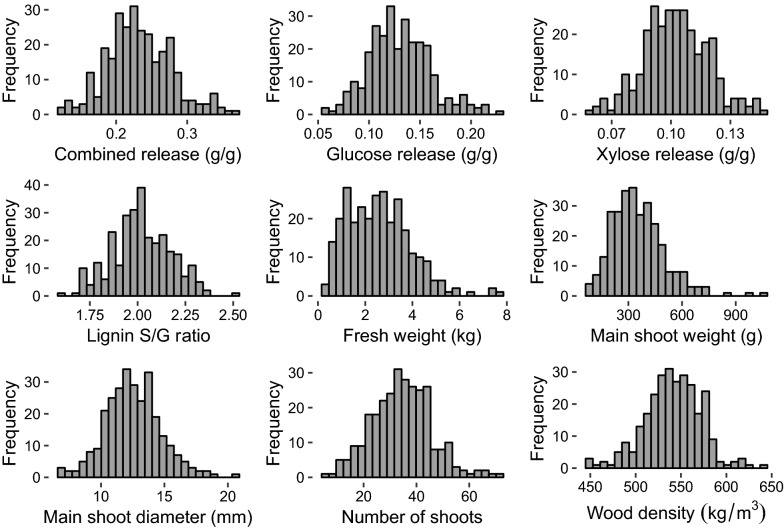
Histograms showing the variation of clonal means in the dataset

For the main shoot of each plant, fresh weight (MSW) and wood density were measured. Whole plant fresh weight (FW) and number of shoots on the plant (NSh), which were recorded in 2017 for an earlier study [16], were also included in analyses within the present study (Fig. 1; Table 1).

**Table 1:** Means of all clones, estimates of chip heritability (*h*^2^), and chip-additive genetic and residual coefficients of variation (CV_a_ and CV_e_) for the traits evaluated herein

Trait	Mean	*h*^2^ (95% CI)	CV_a_ (%)	CV_e_ (%)
Combined release (g/g biomass)	0.23	0.26 (0.19–0.34)	13.7	22.9
Glucose release (g/g biomass)	0.13	0.29 (0.21–0.36)	16.7	26.4
Xylose release (g/g biomass)	0.10	0.23 (0.16–0.30)	10.8	19.6
Lignin S/G ratio	2.02	0.42 (0.35–0.49)	6.3	7.4
Plant fresh weight (kg)	2.58	0.35 (0.27–0.42)	40.7	56.0
Main shoot weight (g)	355.3	0.29 (0.22–0.37)	29.3	45.3
Main shoot diameter (mm)	12.6	0.28 (0.21–0.35)	12.3	19.7
Number of shoots	34.5	0.38 (0.31–0.45)	25.4	32.4
Wood density	541.1	0.59 (0.53–0.65)	5.3	4.4

Sugar release values after miniaturized high-throughput pretreatment and enzymatic saccharification were evaluated. Considerable variation in sugar release was found in this population. For glucose, the range of clonal means (i.e., mean values for each genotype) was 0.06–0.23 g/g biomass (Fig. 1). For xylose release and combined glucose and xylose release (hereafter referred to as *combined release*), the ranges were 0.05–0.15 and 0.11–0.37, respectively. Lignin S/G ratios, analyzed using py-MBMS, ranged from 1.6 to 2.5 (Fig. 1).

Chip heritability estimates (*h*^2^) ranged from 0.23 for xylose release to 0.59 for wood density (Table 1). Wood density and lignin S/G ratio both displayed relatively high chip heritabilities, although the chip-additive genetic coefficients of variation (CV_a_) for these traits were quite low. In contrast, classical biomass traits such as the number of shoots and plant fresh weight exhibited lower *h*^2^ estimates (0.29–0.38) but considerable CV_a_ estimates (25.4% to 40.7%). For the sugar release traits, glucose release showed both the highest *h*^2^ and CV_a_.

Klason lignin contents were estimated by py-MBMS. The values were initially used for heritability estimation; however, the estimate for this trait was conspicuously low, meriting validation using wet chemistry. Consequently, this trait was excluded from these analyses due to poor correlation with wet chemistry analyses performed on a subset of samples (*R*^2^ = 0.08; Additional file 1: Figure S1). We believe that differences in sample preparation (e.g., inclusion of bark and extractives) may be the reason for these anomalous results, as the method used for Klason lignin determination had been constructed using debarked and extracted material.

### Phenotypic and genetic correlations

In terms of phenotypic correlations (*r*_p_) and chip-additive genetic correlation (*r*_a_), sugar release traits were positively correlated with several measures of biomass yield, especially main shoot weight and main shoot diameter (*r*_p_ = 0.51–0.55, *r*_a_ = 0.56–0.75), with slightly weaker correlations for plant fresh weight (Table 2). Similarly, lignin S/G ratio was also positively correlated with the sugar release traits (*r*_p_ = 0.37–0.41, *r*_a_ = 0.34–0.38). In contrast, the number of shoots (Nsh) exhibited low and, in the genetic case, nonsignificant correlations with sugar release traits (*r*_p_ = 0.14–0.15, *r*_a_ = 0.12–0.20). Wood density was essentially uncorrelated with all other traits, however a weakly positive genetic correlation with xylose release was found (*r*_a_ = 0.28).

**Table 2:** Phenotypic (*r*_p_, below diagonal) and chip-additive genetic correlations (*r*_a_, above diagonal) for all traits

*G *+ *X*^a^	0.99	0.94	0.36	0.55	0.65	0.64	0.16	0.24
**0.99**	*Glu*	**0.87**	**0.34**	**0.49**	**0.58**	**0.56**	0.12	0.21
**0.95**	**0.89**	*Xyl*	**0.38**	**0.61**	**0.73**	**0.75**	0.20	**0.28**
**0.39**	**0.37**	**0.41**	*S/G*	**0.31**	**0.33**	**0.35**	0.20	0.15
**0.39**	**0.37**	**0.39**	**0.22**	*FW*	**0.82**	**0.81**	**0.69**	0.06
**0.54**	**0.52**	**0.54**	**0.29**	**0.66**	*MSW*	**0.96**	**0.32**	0.03
**0.54**	**0.51**	**0.55**	**0.30**	**0.65**	**0.94**	*Dia*	**0.31**	− 0.07
**0.14**	**0.14**	**0.15**	**0.11**	**0.59**	**0.16**	**0.18**	*Nsh*	0.18
**0.17**	**0.14**	**0.21**	**0.13**	**0.13**	**0.15**	**0.12**	**0.13**	*Dens*

Correlations in bold are significant at the Holm-corrected *p*-level of 0.05

*G + X* combined glucose + xylose release, *Glu* glucose release, *Xyl* xylose release, *S/G* lignin syringyl/guaiacyl ratio, *FW* plant fresh weight, *MSW* main shoot weight, *Dia* main shoot basal diameter, *Nsh* number of shoots, *Dens* wood density

^a^The trait G + X is a compound trait, and thus any correlations its constituents are partly autocorrelative

### Influence of population structure on phenotypic traits

Ancestral population structure was evaluated using the genomewide markers and comprised four clusters originating roughly from Sweden, Western Europe, Eastern Europe, and Russia. These ancestral structure components were significantly associated (*p *< 0.05, loglikelihood ratio test) with all studied traits except for xylose release (Table 3). Notably, ancestry to the West European cluster was associated with a 9% increase in glucose release. For biomass growth traits, the Swedish and Russian clusters were associated with higher plant fresh weights, and the West European and Russian clusters had larger main shoots in combination with a lower number of total shoots.

**Table 3:** Effects of ancestral population covariates on phenotypic traits and their statistical significance (*p*, loglikelihood ratio test)

Trait	*p*	Sweden (%)	W Europe (%)	E Europe (%)	Russia (%)
Combined release (g/g)	0.043	− 2	+ 7	− 3	+ 1
Glucose release (g/g)	0.038	− 2	+ 9	− 5	+ 3
Xylose release (g/g)	0.059	− 2	+ 5	− 2	− 1
Lignin S/G ratio	0.031	+ 2	0	− 1	− 5
Plant fresh weight (kg)	0.044	+ 12	− 10	− 3	+ 15
Main shoot weight (g)	0.023	− 7	+ 11	− 6	+ 46
Main shoot diameter (cm)	0.024	− 1	+ 5	− 4	+ 18
Number of shoots	0.002	+ 18	− 18	+ 3	− 48
Wood density (kg/m^3^)	0.006	− 1	− 1	+ 2	− 10

The four detected population clusters originated roughly from Sweden, Western Europe, Eastern Europe, and Russia

### Genomewide association study

One marker associated with number of shoots was identified below the Holm-adjusted < 0.05 significance level, while six other markers were associated at the suggestive *p*-level of < 0.2 (Table 4). All these associations individually explained only a limited amount of the phenotypic variation (*R*^2^ = 2.1–2.4%). One notable association was between glucose release and the marker S1_306291149 (*p* = 6.16 × 10^−6^, Holm-adjusted *p* = 0.12). This particular marker was located on chromosome 15 at position 19051974 in a noncoding area of the genome and explained 2.4% of the phenotypic variation of glucose release. The effect of the genotype class homozygous for the rare C allele, found in five individuals with low coancestry (*θ* = 0.044–0.087), was a 27% increase in glucose release, whereas the other genotype classes of this SNP exhibited considerably smaller effects.

**Table 4:** Details of genetic markers identified by genomewide association mapping

Marker	Chr	Pos	*p*	Adj. *p*	*R* ^2^	Genotypes	Effects (%)	*n*	Genes in region
Glucose release									
S1_306291149	15	19051974	6.16 × 10^−6^	0.12	0.024	TT	+ 3	195	SapurV1A.0168s0180 (Cytochrome P450 family protein) SapurV1A.0168s0190 (50S ribosomal protein L21)
						TC	− 8	91	
						CC	+ 27	5	
Main shoot weight									
S1_193869984^a^	9	3038726	2.84 × 10^−6^	0.06	0.022	AA	− 1	248	SapurV1A.0128s0200 (PWI domain protein) SapurV1A.0128s0210 (NAD(P)H-binding family protein)
						AT	− 4	39	
						TT	+ 110	4	
Main shoot diameter									
S1_144195901	6	12104112	4.70 × 10^−6^	0.09	0.022	CC	− 2	238	SapurV1A.0141s0240 (DVL9)
						CT	+ 10	44	
						TT	+ 4	9	
S1_90313329^a^	3	16251727	6.26 × 10^−6^	0.12	0.022	CC	0	253	SapurV1A.0081s0090 (F-box protein interaction domain protein) SapurV1A.0081s0100 (CMT-type cytosine DNA-methyltransferase 3b, putative)
						CT	− 4	30	
						TT	+ 40	8	
S1_99038120	4	8305537	6.67 × 10^−6^	0.13	0.022	AA	+ 3	193	SapurV1A.0985s0030 (heat shock protein-binding protein)
						AC	− 3	74	
						CC	− 15	24	
Number of shoots									
S1_398281479	1236	49140	2.29 × 10^−6^	0.04	0.024	CC	+ 3	168	SapurV1A.1236s0060 (gamma-soluble NSF attachment protein)
						CA	− 10	98	
						AA	+ 18	25	
S1_226299259	11	3701201	9.77 × 10^−6^	0.19	0.021	AA	+ 2	202	SapurV1A.0032s0300 (Est1 DNA/RNA-binding domain protein)
						AG	0	75	
						GG	− 37	14	

For each marker, the most common genotype class is listed first. All markers are of type SNP. Genes within ± 3 kbp of the marker, according to the *S. purpurea* v1.0 genome assembly, are included in the table

^a^The rare-allele homozygotic class for this marker is to a large degree comprised of a set of very closely related individuals. See main text

In total, six marker–trait associations were identified for the non-sugar release traits, with three found for main shoot diameter, one for main shoot weight, and two for the number of shoots. No marker–trait associations were identified for lignin S/G ratio and wood density. Two of the markers, S1_193869984 and S1_90313329, associated with main shoot weight and main shoot diameter, respectively, exhibited a strong recessive effect heavily dependent on a particular group of five rare-allele homozygotes with uniquely high coancestry (*θ* > 0.6) and which all originated east of the Ural mountain range. Therefore, these particular associations should be interpreted with skepticism as they may be due to a specific population substructure that our analysis, despite our efforts, was unable to account for. This particular phenomenon was also seen in a previous GWAS on this population [16].

## Discussion

Developing new low-recalcitrance feedstock varieties through breeding represents one method for improving the financial feasibility of lignocellulosic biorefineries. Here, we present the first survey of sugar release traits, indicative of biomass recalcitrance, in a natural population of *S. viminalis* clones including estimates of genetic parameters for several traits relevant to breeding for improved biorefinery performance in this species. We have also investigated the genetic architecture underlying the observed trait variation, as well as correlations with other traits, including biomass yield parameters and lignin composition.

The range of sugar release in this population was large, with clonal means exhibiting a fourfold difference in glucose release between the extreme clones. This variation is larger than that reported in commercial *Salix* [14], winter wheat [17], and barley [18] varieties, but smaller than that found in natural *Populus* clones [19]. It should be noted that both absolute sugar yields as well as ranges depend on several factors including sample preparation, pretreatment parameters, and the type and dose of enzyme used. In this study, pretreatment and saccharification parameters were optimized for maximizing the variation rather than yields, and results may thus not be directly comparable between studies nor reflect real-world performance.

All traits showed appreciable heritability estimates (*h*^2^ > 0.2) confirming a certain degree of genetic control over the phenotypic variation. In terms of coefficients of genetic and residual variation (CV_a_ and CV_e_), whole plant fresh weight and main shoot weight exhibited the greatest variation. On the other hand, wood density and S/G ratio showed the smallest coefficients of genetic variation (5.3% and 6.3%, respectively) although their corresponding heritability estimates were the highest among the traits studied. This pattern of high *h*^2^ paired with a low CV_a_ appears to be commonly reported for structural wood traits in several conifer species [20–23], and, in addition, it also agrees with the low CV_a_ (6.2%) and high heritability (0.56) for density, calculated from genetic parameters estimated in a wood study in *Populus trichocarpa* [24]. Glucose release exhibited larger additive genetic variation than xylose release, an important result for feedstock improvement as glucose is the more valuable carbohydrate for microbial conversion. Chip heritability estimates for the sugar release traits evaluated in this work (0.23–0.29) were largely in line with those found by other researchers [17, 18, 25, 26] and suggest that it is possible to breed for lower-recalcitrance *Salix* varieties. These observations demonstrate the substantial variation found in natural *Salix* clones sampled across large parts of Europe, which, together with the moderate heritability estimates, constitute a valuable basis for selective breeding.

Strong correlations (*r*_p_ = 0.52–0.54, *r*_a_ = 0.58–0.73) were observed between main shoot weight and the sugar release traits, closely mirrored by main shoot diameters. The S/G ratio was also positively correlated with sugar release (*r*_p_ = 0.34–0.41, *r*_a_ = 0.34–0.38). Biomass yield is generally found to be positively correlated with cellulose and negatively with lignin content in both *Salix* [27, 28] and *Populus* [29, 30], and, moreover, smaller shoots with lower wood/bark ratios will have lower sugar contents due to lower carbohydrate contents in the bark [31]. Although growth traits are more easily measurable than S/G ratios and thus easier to use in selective breeding, their effect on sugar release may be at least partly due to higher proportions of cellulose and hemicellulose, confounding the correlation with recalcitrance. On the other hand, S/G ratios may influence recalcitrance through modulation of a specific recalcitrance factor, namely lignin structure [5]. Thus, while both traits may influence the same objective outcome, i.e., higher sugar yields per mass unit of biomass, a synergistic effect could possibly be gained by selecting for both traits. However, it is worth noting that no correlations were found between saccharification potential and growth traits in a natural population of *Populus nigra* [26], although the very mild pretreatment and saccharification conditions used in that study, developed for *Arabidopsis* [32], may have been insufficient for woody material. Furthermore, although the correlation between S/G ratio and sugar release was positive and statistically significant for this population and specific set of assay conditions, further validation of this finding is warranted before using it in a selection program due to the heterogeneity of results reported in the literature [5].

There were only weak positive correlations between wood density and sugar release traits, in both the phenotypic and genetic sense, with most genetic correlations being statistically nonsignificant. Although a strong positive correlation between density and ethanol production from *Salix* was previously reported [15], the number of clones evaluated in the present work far exceeds that of the previous study. As wood density was genetically uncorrelated, and phenotypically even weakly positively correlated with all measures of biomass growth, this highly heritable trait may represent a viable breeding target for reducing the bulk of *Salix* biomass without impacting sugar release or yield.

In terms of marker–trait associations, S1_306291149 was associated with glucose release at the suggestive significance level (adjusted *p* = 0.12) indicating that this trait could be improved by selecting clones homozygous for the rare C allele at this marker. The marker was located near the gene SapurV1A.0168s0180 according to the *S. purpurea* genome assembly. This gene encodes the protein CYP89A2, a member of the cytochrome P450 family of enzymes, a group of enzymes which catalyzes a wide range of redox reactions and are involved in virtually all secondary metabolic pathways including lignin, terpene, and plant hormone biosyntheses [33, 34]. We are not aware of any literature investigating the function of this gene, or of its *Populus* or *Arabidopsis* orthologs, and thus cannot speculate on the possible mechanism of action in the influence of this gene on saccharification yields.

Given the limited percentage of phenotypic variance explained by the marker–trait association discussed above (*R*^2^= 2.4%) and the lack of other significant associations it appears likely that biomass recalcitrance is a highly polygenic trait, especially given that heritability estimates for sugar release traits were still appreciable (*h*^2^ > 0.2). Biomass recalcitrance is indeed a complex phenomenon dependent on several gross compositional (e.g., cellulose and lignin content), chemical (e.g., lignin composition), as well as micro- and ultrastructural features affecting cellulose accessibility [3]. In a two-stage approach directed at a genetic region known to be involved in cell wall architecture in *Populus*, Muchero and coworkers managed to link several SNP markers to sugar yields and cell wall chemistry [19], revealing multiple diverse genes linked to cell wall composition, sugar yields, or both. However, more general GWAS approaches have generally failed to find markers significantly associated with sugar yields (e.g., [18, 25, 26]), a result that is mostly mirrored in the present study. The poor ability of GWAS to resolve marker–trait correlations for highly polygenic traits is a well-known limitation of this methodology [35]. Further, the inherent variability of high-throughput assays, such as the saccharification protocol used in this study, increases the level of noise in the data, making power-demanding statistical analyses, such as GWAS, challenging. However, we were still able to perform a robust quantitative genetic analysis of recalcitrance traits that is relevant for plant breeding purposes.

It is worth noting that only the main shoot of each plant was used for the sugar release assay, whereas whole plant sugar yields will depend on the sum of yields of all shoots. The finding that main shoot weights and diameters were positively correlated with sugar release suggests that accumulation of biomass in fewer, larger shoots may represent a component of the *Salix* ideotype for optimal per-hectare sugar yields (i.e., the product of biomass and sugar yield). Out of the four population clusters, this growth pattern was mainly observed in the Russian cluster (Nsh − 48%, MSW + 46%, Dia + 18%) but to a certain extent also in the West European cluster (Nsh − 18%, MSW + 11%, Dia + 5%), reflecting potential reservoirs of genetic variation for use in breeding programs. Furthermore, for glucose and total sugar release, population structure components exerted a significant effect indicating that clones belonging to the West European population cluster were associated with 9% higher glucose release, despite main shoot weight being only slightly elevated and S/G ratio being at the mean. Thus, the West European population cluster may harbor other recalcitrance factors not evaluated in this study.

## Conclusions

Natural populations of *S. viminalis* harbor considerable variation in sugar release traits, constituting a valuable resource for breeders looking to produce new *Salix* varieties for biorefinery use. This work represents the first large-scale survey of recalcitrance in this commercially relevant bioenergy crop species. Estimates of chip heritability and genetic coefficients of variation for these traits suggest that breeding toward this goal is feasible. Main shoot weights, shoot diameters, and lignin S/G ratios were positively correlated with sugar release and may thus represent indirect measurement traits to use in the selection and improvement of sugar release, although inconsistencies in the literature suggest that further validation of the correlation between S/G ratio and recalcitrance as a proxy trait needs to be examined further. Finally, one marker (S1_306291149) was indicated to be associated with glucose release, meriting further investigation.

## Materials and methods

### Plant material and field experimental design

The plant material used in this study originates from a previously established population of 323 genetically distinct *S. viminalis* accessions. Each accession was vegetatively propagated (i.e., cloned) and planted in a completely randomized block design encompassing six blocks in Pustnäs, Uppsala, Sweden (59º48′N, 17º39′E). Details of the population have been provided in earlier publications [10, 36]. In brief, clones were collected in the wild throughout Europe and Russia, in locations spanning latitudes from 48.1°N to 62.4°N and longitudes from 4.8°W to 104.3°E. The field experiment was initiated in 2009.

### Harvesting and density measurements

The plants were harvested winter 2016/17 having 2-year old shoots and 8-year old roots. Plants from four out of six blocks were used for this study. Number of shoots and fresh weight was recorded for each plant in the experiment. The highest shoot of each plant was selected and harvested separately, and the weight and diameter of the shoot was measured.

From each individual shoot, an approximately 25 cm long piece from the bottom part of the shoot was cut and separated into two subsamples. The first subsample (bottom part, approximately 20 cm long) was saved for chemical analyses, while the second subsample (approximately 5 cm long) was used for density measurements. Each such sample was debarked and dried in an oven at 103 ± 2 °C. After drying, the absolute dry weight of the sample was recorded and immediately the sample’s absolute dry volume measured by immersion in water. The two measurements were used to calculate the absolute dry density of the wood.

### Sample preparation for sugar release and py-MBMS assays

After removing the bottom part of the shoot as described above, the remainder of the main shoot of each plant (including bark) was coarsely chipped using a compost grinder (Viking GE 150, Viking GmbH, Langkampfen, Austria), and stored in air-tight bags at − 4 °C. Aliquots of approx. 5 g were dried for 6 h at 60 °C. After drying, samples were milled using a Wiley Mini-Mill (Thomas Scientific, Swedesboro, NJ, USA) and passed through a 20-mesh sieve. The ground material was stored in antistatic sample bags at room temperature.

### High-throughput pretreatment

A high-throughput pretreatment (HTP) assay was performed at the National Renewable Energy Laboratory (NREL) in Golden, CO, USA. This assay was conducted largely as described by Decker et al. [37], using 5 mg of unextracted biomass per reaction and 250 μl deionized water as catalyst in Hastelloy 96-well plates. As the material was collected after senescence in the field, and thus could be expected to contain very low amounts of starch, the destarching step [38] was omitted. All samples were pretreated at 180 °C for 17.5 min corresponding to a severity factor (log_10_ *R*_0_) of 3.6, calculated using the formula:

$$R_{0} = t \times e^{{\left( {\frac{T - 100}{14.75}} \right)}},$$

where *t* is the time in minutes and *T* is the temperature in °C. Each sample was pretreated in triplicate, and 24 samples were loaded on each 96-well plate. BESC standard poplar was used for the controls, and each plate contained eight controls and four blanks.

### Enzymatic hydrolysis

For the enzymatic hydrolysis, Accellerase 1500 (78.1 mg BCA protein/ml; lot 1662334068; DuPont, Palo Alto, CA, USA) and Multifect xylanase (45.4 mg BCA protein/ml; lot 301-04296-205; DuPont, Palo Alto, CA, USA) were used. The loadings were 80 mg and 20 mg/g biomass for the Accellerase and Multifect enzymes, respectively. Hydrolysis was conducted at 50 °C for 70 h, with 0.02% sodium azide in 140 mM pH 5.0 citrate buffer. Glucose and xylose release values were then measured using GOPOD and XDH enzymatic assays according to the manufacturer’s instructions (Megazyme, Bray, Ireland).

### Enzyme dosage and pretreatment severity assay

To determine suitable pretreatment and saccharification parameters to differentiate recalcitrance levels between samples, defined as those maximizing the spread of the data, pretreatment conditions and enzyme dosages were determined experimentally. First, a trial was carried out using either 100% Accellerase 1500 or 80% Accellerase 1500 and 20% Multifect xylanase, at two different lengths of pretreatment (12.0 and 17.5 min) and protein loadings (20 and 70 mg protein/g biomass). Severities for these pretreatments were log *R*_0_ = 3.4 and log *R*_0_ = 3.6, respectively. The results of this trial indicated that the pretreatment of log *R*_0_ = 3.6 and the 80/20 mixture was most appropriate (Additional file 1: Figure S2). A second trial, using a larger number of samples and the loadings 70, 100, 125, and 150 mg/g biomass of the same Accellerase/Multifect mixture, pretreated at log *R*_0_ = 3.6, identified the 100 mg enzyme/g biomass loading as optimal (Additional file 1: Figure S3).

### Pyrolysis-molecular beam mass spectrometry (py-MBMS)

Nonpretreated, unextracted, milled samples were analyzed using py-MBMS according to methods previously described [6], using helium as a carrier gas, flow set to 0.9 l/min, furnace temperature 500 °C, and interface temperature 350 °C. Mass-to-charge (*m/z*) values were collected over the range of 30–450. Each sample was analyzed in duplicate. Lignin S/G ratios were calculated relative to a standard with an S/G ratio of 1.7, as determined by thioacidolysis [39].

### Biomass compositional analysis

For validation of Klason lignin estimates, a subset of 17 samples were chosen at random from the material used for density measurements. The samples were milled to pass a 40-mesh screen, and analyzed for acid soluble lignin (ASL), acid insoluble lignin (AIL), and monosaccharides according to the procedure of Sluiter et al. [40]. The ASL was determined using a Hitachi U-2910 spectrophotometer (Hitachi, Tokyo, Japan) with an absorptivity of 110 l/g/cm at a wavelength of 205 nm. The monomeric carbohydrates were determined using a Chromaster high-performance chromatography (HPLC; Hitachi, Tokyo, Japan) system equipped with an evaporative light scattering detector (ELSD-90; VWR International GmbH, Darmstadt, Germany), and a Metacarb 87P column (300 mm × 6.5 mm; Santa Clara, CA, USA) with a guard column (Metacarb 87P 50 mm × 4.6 mm). The ELSD-90 was operated at 50 °C, 2.5 bars, and N_2_ was used as the nebulizing gas. The sugars were eluted using ultrapure water as a mobile phase at a constant flow rate of 0.5 ml/min and column temperature of 85 °C.

### Genotyping-by-sequencing and marker generation

From clones of the study population, young leaves were sampled, and DNA was extracted according to a protocol described in [41]. DNA extracts were then genotyped by sequencing (GBS) [42] at the Genomic Diversity Facility, Cornell University, Ithaca, NY. DNA was digested by the ApeKI restriction endonuclease ligated to sample-specific barcode adapter sequences and then sequenced on an Illumina Next-generation sequencing (NGS) platform (Illumina Inc., San Diego, CA). Sequence reads and polymorphisms were provided by running the TASSEL-GBS analysis pipeline [43] using the available genome sequence of the close relative *Salix purpurea* as a mapping reference (*Salix purpurea* v1.0 [44], DOE-JGI, https://phytozome.jgi.doe.gov/pz/portal.html#!info?alias=Org_Spurpurea). For the 291 clones for which DNA sampling, preparation, and genotyping were successful, 1,555,795 sequence sites showing polymorphisms (single-nucleotide polymorphisms, SNPs; and single-nucleotide indels, SNIs) were identified and merged by TASSEL 4.3.7. The merged genotype data are stored at a publicly available repository [45].

For putative polymorphic sites, diploid genotypes were called according to the maximum likelihood [46] requiring at least 5 reads per site and clone for any definitive call to be made. The GBS polymorphism data were thereafter merged with older genotyping data (1290 SNPs) previously developed for the study population [34]. Polymorphic sites were then filtered using VCFtools 0.1.12b based on data completeness and on minor allele frequency (MAF) depending on downstream use (see [16] for details). To reduce the number of sites produced by erroneous merging of paralogous sequences, all polymorphisms with an apparent heterozygosity above 70% were removed using a custom Perl script.

### Population structure and kinship

In order to take into account effects of population structure and clone relatedness, a kinship matrix (**K**) was estimated in accordance with [47] using a 19,243 marker dataset where included sites were required to have > 95% called genotypes and a minor allele frequency (MAF) > 1%. In addition, population ancestry was investigated by applying an admixture model with correlated allele frequencies within population using the software STRUCTURE version 2.3.4 [48–50] which uses a fifth of the markers (3848) used for estimating **K**. Based on this assessment, four population clusters—Swedish, Russian, East European, and West Europan—were identified (see [10, 16] for details).

### Statistical analyses

Statistical analyses were performed using R version 3.5.1 [51], and figure plots were created using ggplot2 version 2.2.1 [52]. Measurement data for all phenotypic traits were subjected to quantitative genetic analysis using the R package sommer version 3.6 [53], first applying the following univariate mixed model to each phenotypic trait (**y**) separately:

$${\mathbf{y}} = {\mathbf{Xb}} + {\mathbf{Zu}} + {\mathbf{e}},$$

where **b** is the vector of fixed intercept and block effects, **u** is the random chip-additive genetic effects of each clone, and **e** is the random residual, and where the design matrices **X** and **Z** link the respective block and chip-additive genetic effects to their observations. All effects were assumed to be independent except for the chip-additive genetic effects which were assumed to follow the structure $Var\left( {\mathbf{u}} \right) = 2\sigma_{A}^{2} {\mathbf{K}},$ where $\sigma_{a}^{2}$ is the chip-additive genetic variance. Narrow-sense chip heritabilities (*h*^2^) and corresponding additive genetic and residual coefficients of variation (CV_a_ and CV_e_) were then calculated by means of the R package heritability version 1.2 [38] using the equations:

$$h^{2} = \frac{{\sigma_{\text{a}}^{2} }}{{\sigma_{\text{a}}^{2} + \sigma_{\text{e}}^{2} }} \;\;\;{\text{CV}}_{\text{a}} = \frac{{\sigma_{\text{a}} }}{\mu } \;\;\;\;{\text{CV}}_{\text{e}} = \frac{{\sigma_{\text{e}} }}{\mu },$$

where $\sigma_{\text{e}}^{2}$ and *µ* represent the residual variance and the field trial mean, respectively. Effects of population structure on traits were evaluated using the mixed model above but including the population structure covariates (**Fq**) as fixed effects. The statistical significance of the structure covariates was assessed using a loglikelihood ratio test (*χ*^2^-distribution with 3 degrees of freedom), comparing the models with and without population structure covariates. Effects were calculated against the weighted mean for all population covariates.

Genetic correlations were calculated by fitting two traits simultaneously in bivariate mixed models but using the same linear model as previously described. Statistical significances of the genetic correlations were evaluated by a loglikelihood ratio test (*χ*^2^-distribution with 1 degree of freedom), comparing a model where the chip-additive genetic variance–covariance matrix was considered to be unstructured (no components constrained) with a null-hypothesis model where the matrix was considered to be block-diagonal (covariance component locked at zero). Adjustments for multiple significance testing of genetic correlations were performed using the p.adjust() function in R, using the method “holm” [54]. Phenotypic correlations were tested for statistical significance using the method cor.test() in R, again multiple-correction adjusted using the “holm” method.

### Genomewide association analysis

For genomewide association analyses, all markers that were found to be biallelic, to be nonredundant, to have > 75% called genotypes and a MAF > 5% were included in the association analysis (totaling 17,853 SNPs and 1558 SNIs). Missing genotype calls were imputed using the LD-kNNi method as formulated in the software *LinkImpute* [55] and where the genotypes of neighboring markers in close LD with the marker to be imputed (< 10 Mb distance) were used as support. Associations were calculated using TASSEL 5.2.43 [56] using the full model including kinship and population structure:

$${\mathbf{y}} = {\mathbf{Xb}} + {\mathbf{Fq}} + {\mathbf{Sg}} + {\mathbf{Zu}} + {\mathbf{e}},$$

where **g** is the vector of genotype marker effects, and the design matrix **S** constitutes the individual genotype for each marker analyzed separately. Genotype effects for each marker were considered to be independent factors of the form **g** = [*g*_*AA*_ *g*_*Aa*_ *g*_*aa*_]^*T*^ for common allele homozygotes (*AA*), heterozygotes (*Aa*), and rare-allele homozygotes (*aa*), respectively. All other effects were as described previously. All biological replicates were used in a single-step analysis (i.e., no clonal estimators/predictors were used as intermediaries). Adjustments for multiple significance testing of marker–trait association *p*-values were again performed using the p.adjust() function in R, using the method “holm.” Markers with adjusted *p* values < 0.2 are reported herein. To calculate relative marker effects as percentages, effects as reported by TASSEL were first adjusted by subtracting the weighted mean of effects. The adjusted means were then divided by the population mean for the trait.

## Additional file

**Additional file 1: Figure S1.** Estimates of Klason lignin from py-MBMS versus values obtained by wet chemistry. **Figure S2.** Results of initial test runs to assess optimal pretreatment severity and enzyme loading. **Figure S3.** Results of second test run to better assess optimal enzyme loading.

## Data Availability

Genotypic data for the study population generated by the Genotyping-by-sequencing (GBS) process are publically available at the Zenodo repository (http://doi.org/10.5281/zenodo.2607520). All other data are available on request.
