# Supplementary material for: Genetic variation of biomass recalcitrance in a natural Salix viminalis (L.) population
Source: Biotechnol Biofuels. 2019 Jun 3;12:135. doi: 10.1186/s13068-019-1479-7 (PMC6545741; doi:10.1186/s13068-019-1479-7)
Supplement: Supplementary file 1 — Additional file 1: Figure S1. Estimates of Klason lignin from py-MBMS versus values obtained by wet chemistry. Figure S2. Results of initial test runs to assess optimal pretreatment severity and enzyme loading. Figure S3. Results of second test run to better assess optimal enzyme loading. [file 13068_2019_1479_MOESM1_ESM.docx]

Figure S1. Estimates of Klason lignin from py-MBMS versus values obtained by wet chemistry.

Figure S2. Results of initial test runs to assess optimal pretreatment severity and enzyme loading. Legend: C, Accellerase 1500 only; X, Accellerase 1500 and Multifect Xylanase 80/20% mixture; L, low enzyme dosage; H, high enzyme dosage; 12, 12.0 min pretreatment time; 17, 17.5 min pretreatment time.

Figure S3. Results of second test run to better assess optimal enzyme loading. Treatments A070–A150 correspond to Accelerase 1500 and Multifect Xylanase 80/20% mixtures at dosages of 70–150 mg/g biomass. A100 corresponds to the parameters used for the main study.
